# Bridging cognition and action: executive functioning mediates the relationship between white matter fiber density and complex motor abilities in older adults

**DOI:** 10.18632/aging.204237

**Published:** 2022-08-22

**Authors:** Caroline Seer, Hamed Zivari Adab, Justina Sidlauskaite, Thijs Dhollander, Sima Chalavi, Jolien Gooijers, Stefan Sunaert, Stephan P. Swinnen

**Affiliations:** 1 Movement Control and Neuroplasticity Research Group, Department of Movement Sciences, KU Leuven, Leuven, Belgium; 2 KU Leuven Brain Institute (LBI), KU Leuven, Leuven, Belgium; 3 Murdoch Children’s Research Institute, Melbourne, Australia; 4 Department of Imaging and Pathology, KU Leuven and University Hospital Leuven (UZ Leuven), Leuven, Belgium

**Keywords:** aging, executive functions, fixel-based analysis, motor control, white matter connectivity

## Abstract

Aging may be associated with motor decline that is attributed to deteriorating white matter microstructure of the corpus callosum (CC), among other brain-related factors. Similar to motor functioning, executive functioning (EF) typically declines during aging, with age-associated changes in EF likewise being linked to altered white matter connectivity in the CC. Given that both motor and executive functions rely on white matter connectivity via the CC, and that bimanual control is thought to rely on EF, the question arises whether EF can at least party account for the proposed link between CC-connectivity and motor control in older adults. To address this, diffusion magnetic resonance imaging data were obtained from 84 older adults. A fiber-specific approach was used to obtain fiber density (FD), fiber cross-section (FC), and a combination of both metrics in eight transcallosal white matter tracts. Motor control was assessed using a bimanual coordination task. EF was determined by a domain-general latent EF-factor extracted from multiple EF tasks, based on a comprehensive test battery. FD of transcallosal prefrontal fibers was associated with cognitive and motor performance. EF partly accounted for the relationship between FD of prefrontal transcallosal pathways and motor control. Our results underscore the multidimensional interrelations between callosal white matter connectivity (especially in prefrontal brain regions), EF across multiple domains, and motor control in the older population. They also highlight the importance of considering EF when investigating brain-motor behavior associations in older adults.

## INTRODUCTION

Aging is a multifaceted process that can affect individuals differently. On a group level, older age is associated with changes in motor performance that may potentially impact healthy and active living [1]. In particular, successful coordination of both hands is a crucial motor skill that has relatively consistently been found to be affected by aging, with possible consequences for functional independence [2, 3]. Among several factors [1], neural and cognitive aspects of aging have been considered as contributors to decrements in bimanual movement control. That is, both an age-associated decrease in brain white matter connectivity and an age-related decline in executive functioning (EF) are thought to give rise to functional impairments. These perspectives are not mutually exclusive but represent different levels to account for variations in bimanual coordination at older age.

Widespread changes in white matter connectivity between brain areas have been reported at older age [4–7]. Specifically, age-associated alterations in the micro- and macrostructure of the corpus callosum (CC) have been proposed to contribute to the observed age-related decline in bimanual control as detected in laboratory settings [8–14]. This suggests that successful coordination of both hands depends on intact white matter connections via the CC—the major neural pathway that connects homologous cortical areas of the two cerebral hemispheres [3, 12, 15–20].

Besides motor functions, age-related differences in cognitive functions (including EF) have been reported [21–23]. Executive functions are a set of generic higher-order mental processes that are thought to guide and control lower-level mental processes to enable successful goal-directed behavior [24, 25]. Earlier work has suggested that age-related changes in EF may contribute to or exacerbate age-related motor difficulties [26–28], potentially because older adults rely on executive functions to a larger extent than young adults when performing complex motor tasks [29, 30]. Similar to motor functions, EF were shown to be associated with white matter tracts connecting multiple brain areas, including prefrontal, fronto-parietal, and subcortical areas [31]. Damage to the white matter microstructure of this executive network has been interpreted to result in network “disconnection” and—consequentially—functional decline [32, 33]. Accordingly, age-associated changes in EF have been linked to decreased white matter connectivity [34–41]. More specifically, the importance of transcallosal fibers for EF in aging has been highlighted. For instance, older adults’ performance on EF tasks has been shown to be associated with fractional anisotropy [42, 43] and with size measures of the CC [44]. This is in line with the notion that performance of EF tasks recruits a broad bilateral network, including frontoparietal regions of the cerebral cortex [45]. Taken together, the available literature suggests that (1) normal aging is typically associated with declines in white matter connectivity as well as changes in executive and complex motor control, (2) EF aids successful complex motor control, especially in older adults, and (3) callosal white matter connectivity plays a role in both EF and complex motor control. Despite these findings, interrelations between white matter microstructure of the CC, EF, and motor control have not yet been investigated in sufficient detail. However, associations between these three variables seem plausible because microstructural organization of transcallosal fibers connecting prefrontal regions—which are often associated with EF—have been found to play an important role in bimanual motor learning in young adults [18]. With respect to performance in bimanual tasks, we demonstrated that the relationship between age and task performance was mediated by the microstructural properties of white matter tracts passing through the anterior parts of the CC, which connect bilateral prefrontal cortices. All together, these findings point toward the involvement of executive functions in bimanual movement control [14].

Hence, we hypothesized that age-related differences in transcallosal white matter connectivity may contribute to variations in bimanual movement control directly, as suggested by earlier studies, but also indirectly, via decreased EF. Here, we focused on the indirect route via the study of interrelations between EF, bimanual control, and transcallosal white matter connectivity in older adults. Specifically, we hypothesized that individual differences in EF can—at least in part–account for the observed link between white matter connectivity of the CC and bimanual movement control in this population. To investigate this, we combined diffusion MRI (dMRI) data acquisition with the assessment of both executive and bimanual motor performance in a large sample of older adults. Up to 90% of white matter voxels have been estimated to show complex fiber geometries that the traditional diffusion tensor-based approach is unable to resolve [46–48]. To overcome these methodological limitations, in our assessment of white matter connectivity, we relied on a fixel based analysis (FBA) approach, analyzing fiber populations within single voxels (“fixel”) [49, 50]. For the assessment of EF, we utilized a latent variable metric. Specifically, we derived a common EF factor by extracting the shared variance from a comprehensive battery of EF tasks. This approach increases reliability and improves generalizability compared to the evaluation of performance indices from a single EF task [51, 52]. However, to the best of our knowledge, such an approach has not yet been undertaken in the study of cognition-action interactions in relation to white matter connectivity.

## MATERIALS AND METHODS

### Participants

The effective sample size comprised *N* = 84 older adults (52 female, 32 male; 70 right-handed, 4 left-handed, 10 ambidextrous) between 60 and 85 years of age (*M* = 68.06, *SD* = 4.74). All participants had normal or corrected-to-normal vision. Participants were excluded if they reported current intake of psychoactive medication, a current diagnosis of psychiatric/neurological disorder, upper limb injury that would have interfered with the completion of the motor task, and/or MRI contraindications. None of the participants showed signs of mild cognitive impairment, as based on the Montreal Cognitive Assessment (MoCA; *M* = 27.71, *SD* = 1.82, range: 24-30) [53, 54]. The average number of education years was 18.96 (*SD* = 2.72; range: 11-24) and the average level of crystallized intelligence on the Peabody Picture Vocabulary Test (PPVT) was 109.50 (*SD* = 8.76, range: 82-125) [55, 56]. Hence, the individuals enrolled in this study were relatively well functioning in both cognitive and motor abilities. This choice was intended to describe the relationships among white matter measures, EF, and motor performance in the absence of any severe pathology. Participants were recruited from the area of Leuven (Belgium) as part of a larger project [28]. The original sample included 111 older adults. We retained only complete datasets, i.e., those with sufficient quality of dMRI and scores on motor and EF tasks, and therefore excluded 27 datasets, as indicated in Supplementary Figure 1.

The study was reviewed and approved by the Ethics Committee Research UZ/KU Leuven (study number 61577). All participants gave written informed consent to participate and were offered a compensation of € 100. The dataset is openly available on https://www.osf.io/f7nvd/. The code for dMRI processing is based on the general FBA pipeline [49, 50] provided on the MRtrix website [57] (https://mrtrix.readthedocs.io/en/latest/fixel_based_analysis/mt_fibre_density_cross-section.html).

### Procedure

The study protocol consisted of three separate sessions. In the first behavioral session, participants completed background assessments and questionnaires as well as three computerized EF tasks, and were familiarized with the motor task. In the second behavioral session, participants completed six computerized EF tasks and performed the motor task (see [28] for a detailed description). Neuroimaging took place in a separate session. The mean time interval between the first and the third session for an individual was 15.76 days (*SD* = 14.12; range: 2-72).

### Behavioral assessments

### Executive functioning tasks

Participants completed nine neuropsychological tasks across two test days, following a protocol similar to [58]. These tasks covered three key domains of EF, i.e. inhibition (suppressing unwanted actions), shifting (switching between mental operations), and updating (managing working memory content). Every domain was represented by three tasks (inhibition: antisaccade, number Stroop, stop signal; shifting: category switch, color-shape, number-letter; updating: digit span, keep track, spatial 2-back). Tasks were presented in a fixed order to minimize between-subject variability, which would have complicated the latent variable extraction (day 1: stop signal, category switch, digit span; day 2: color-shape, keep track, antisaccade, spatial 2-back, number Stroop, number-letter) [58]. The task parameters and outcome calculations per task are described in detail in [28]. In the context of the present study, these tasks served to derive a measure of general executive abilities. To this end, performance outcomes were calculated based on reaction time or accuracy (see Supplementary Table 1 for a more detailed description). These scores were entered into a confirmatory factor analysis in lavaan 0.6-7 [59], where a common EF factor represented the shared variance by all nine tasks and residual variability from shifting and updating tasks was modeled as “shifting-specific” and “updating-specific” factors [52]. This model fit the data well, χ^2^(21) = 21.60, *p* = .423; comparative fit index (CFI) = .992; root mean square error of approximation (RMSEA) = .02; standardized root mean square residual (SRMR) = .07. Values for CFI > .95, RMSEA < .06, and SRMR < .08 are considered good [60].

The common EF factor score was used as a mediator variable when assessing the interrelations between EF, complex movement control, and white matter microstructure [25, 51, 52]. Investigating a common EF score is particularly useful in the context of our analysis as it takes into account the multifaceted nature of EF by integrating across multiple EF domains. Specifically, the common EF score reflects shared variability across all EF tasks under investigation, therefore providing a comprehensive EF performance index that is not biased toward one EF domain in particular. In addition, assessing EF on the latent variable level improves generalizability and reliability compared to EF analyses on the level of (single or z-averaged) task performance, and it overcomes limitations due to task impurity [52].

### Motor performance tasks

Complex movement control was assessed using a bimanual tracking task (BTT) that determined coordination accuracy. Participants were instructed to track a moving dot on a computer screen by rotating two dials at prescribed frequencies (Supplementary Figure 2). The left hand controlled upward and downward movements of a tracking cursor by clockwise and counterclockwise dial rotations, respectively. Similarly, clockwise and counterclockwise dial rotations with the right hand controlled cursor movements to the right and to the left, respectively. Participants were extensively familiarized with both “straight” (i.e., both hands moving at the same speed without direction changes) and “zigzag” (i.e., both hands moving at the same speed with direction changes in one of the hands) conditions at the end of the two behavioral sessions. At the end of the second behavioral session, participants completed 16 trials of the “straight” condition (not included in these analyses) and 24 trials of the more challenging “zigzag” condition on this task. Specifically, in these zigzag trials, the moving dot followed a zigzag pattern for 15 seconds (for more information on the task details, see [14, 28, 61]). For successful completion of such trials, participants need to perform regular directional switches in the rotational movement of one hand, whereas the other hand’s movement needs to preserve direction. Rotation speed was constant for the two hands. Participants were instructed to keep the cursor as close to the moving target dot as possible at all times. Equal numbers of trials were administered for horizontal (right hand stable, left hand switching) and vertical (left hand stable, right hand switching) zigzag trajectories. The outcome measure was the percent “coverage” of the target line. This calculation was performed such that every sampled cursor point was assigned to the point on the target line with minimal Euclidean distance to the respective cursor position (“covering” that point). The number of unique “covered” points was divided by the total number of points on the target line and multiplied by 100 for every trial. Higher scores hence reflect better coverage of the target line by the cursor and thus higher accuracy [14, 61]. For the current analyses, the average accuracy across all zigzag lines was used as a performance index on the complex motor task [28].

### MRI acquisition

MRI data were acquired on a Philips Achieva 3.0T MRI system equipped with a 32-channel head coil. A high-resolution three-dimensional T1-weighted structural image was collected, using a magnetization-prepared rapid gradient echo (MPRAGE) sequence with the following parameters: TR/TE = 5.6/2.5 ms; flip angle = 8°; voxel size = 0.9 × 0.9 × 0.9 mm^3^; field of view = 256 × 240 × 187.2 mm^3^; 208 sagittal slices; sensitivity encoding (SENSE) = 2; total scan time = ~ 6 minutes. Diffusion MRI data were acquired using a single-shot echo planar imaging sequence with the following parameters: dMRI volumes with b-values = 700 s/mm^2^ (16 gradient directions), 1200 s/mm^2^ (30 gradient directions), and 2800 s/mm^2^ (50 gradient directions); 6 interspersed volumes without diffusion weighting (b = 0 s/mm^2^); flip angle = 90°; phase-encoding direction = posterior to anterior (PA); field of view = 240 × 240 × 140 mm^3^; voxel size = 2.5 × 2.5 × 2.5 mm^3^, TR/TE = 5000/74 ms; multiband factor = 2; SENSE = 2; matrix size = 96 × 94; 56 transverse slices; total scan time = ~ 9 minutes. We also acquired five b = 0 s/mm^2^ images with reversed phase encoding (AP) for the purpose of susceptibility-induced distortion correction.

### Data analysis

### MRI preprocessing

The MRtrix3 [57] standard FBA procedure, described in detail elsewhere [50], was performed on dMRI data. This procedure included: (1) denoising [62], (2) Gibbs unringing [63], (3) corrections for eddy, motion, and susceptibility induced distortions [64–66], (4) 3-tissue (representing single-fiber white matter, grey matter and CSF) response functions estimation [67], (5) upsampling to 1.25 mm^3^ [68, 69], (6) brain mask estimation, (7) multi-tissue constrained spherical deconvolution (CSD) [70] using the averaged (across all subjects) response functions for each tissue type to estimate white matter fiber orientation distribution (FOD), (8) joint bias field correction and global intensity normalization of white matter FOD in log-domain [71], (9) generation of a study-specific population FOD template to which each participant’s FOD image was nonlinearly registered [72, 73], (10) segmentation of the warped FOD to obtain fixels and their apparent fiber density (FD), (11) fixels reorientation, (12) segmentation of the FOD template and assigning the FD value from each participant’s fixel to the corresponding fixel in the template, (13) calculating the logarithm of fiber cross-section (FC) for each fixel from the subject to template warp, and (14) multiplying FD and FC to obtain the FDC value for each fixel (see [50] for details on this pipeline).

The latter metrics and their interpretation have been originally described by [49] and are briefly summarized here. Apparent FD relates to the volume of the intra-cellular compartment of axons oriented along a particular direction, i.e., it relates to the axonal microstructure. FC measures the cross-section of the axon bundle, i.e., it relates to the macrostructure (morphological properties). Finally, FDC incorporates the combined effect of FD and FC [50].

### Corpus callosum (CC) tract-specific analyses

**Figure 1:**
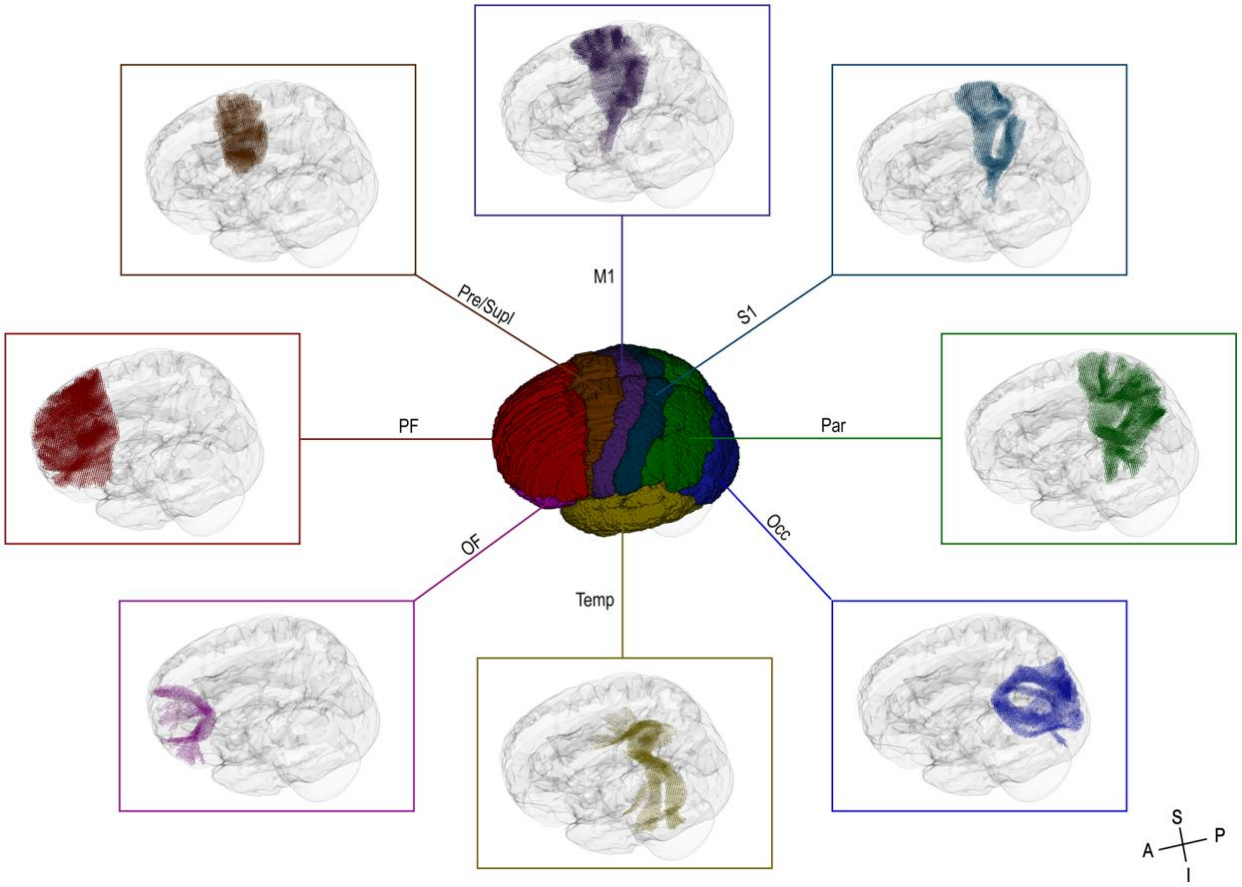
**3D-view of the cortical regions of interest (ROIs) used for the targeted CC tractography (center) and the assigned fixels of interest (surround).** OF = orbitofrontal cortex, PF = prefrontal cortex, Pre/Supl = premotor and supplementary cortex, M1 = primary motor cortex, S1 = primary sensory cortex, Par = parietal cortex, Occ = occipital cortex, Temp = temporal cortex, A = anterior, P = posterior, S = superior, I = inferior.

Similar to the described paradigms by [74, 75], the subdivisions of the CC were delineated based on structural connectivity to functionally distinct occipital (Occ), parietal (Par), temporal (Temp), primary sensory (S1), primary motor (M1), premotor and supplementary (Pre/Supl), prefrontal (PF), and orbitofrontal (OF) cortical regions (Figure 1). Except for the OF, the regions of interest (ROIs) were obtained from the MNI-maxprob-thr25-1mm atlas in FSL [76], and the Human Motor Area Template [77]. To distinguish the OF from PF, an axial slice at the level of the inferior edge of the splenium was used, following the guidelines by [75]. All ROIs were transferred to the study-specific FOD template following linear registration of the FSL HCP1065 standard-space fractional anisotropy (FA) image to the study-specific white matter FOD template. Probabilistic streamline tractography between homologous ROIs was performed using the study-specific FOD template and the 2^nd^-order integration over FOD algorithm [78]. To restrict streamlines to those passing only through the CC, appropriate CC inclusion and midline exclusion masks were manually drawn on the white matter FOD template. Furthermore, to prevent excessive dispersion of streamlines leading to large tract overlap, the ROIs not involved in the active tracking (i.e., those related to other pairs of endpoints or CC segments) were used as exclusion masks [20, 79]. For each CC-tract of interest, one percent of the total number of streamlines was discarded. The remaining streamlines were used to find the corresponding template fixels and to calculate the mean FD, FC, and FDC (Supplementary Table 2). To account for the overall head size, the total intracranial volume (TIV) was obtained from T1-weighted images using the standard pipeline of FreeSurfer [80].

### Statistical analysis

Statistical analyses were performed in SPSS 27 (IBM, Armonk, NY). Relationships between (1) white matter measures (FD, FC, FDC) and motor performance, (2) white matter measures and EF performance, and (3) EF and motor performance were tested using regression analysis and complete standardized regression coefficients (β) are reported. For the relationships involving white matter measures (i.e., (1) and (2)), the TIV was used as a nuisance covariate, as FC (and FDC) are sensitive to the macroscopic volume of the brain. Since 24 different models (eight CC-tracts of interest [Occ, Par, Temp, S1, M1, Pre/Supl, PF, OF] x three outcome metrics [FD, FC, FDC]) were tested, *p*-values were multiplied by 24 to account for multiple comparisons based on the Bonferroni procedure. Thus, the statistical significance was set to *p*_Bonferroni-corrected_ < 0.05.

### Mediation analysis

A widely used mediation model [81] was then applied to examine the hypothesis that motor control (as indicated by BTT performance, corresponding to the “Y” or outcome variable) is related to white matter micro- and/or macrostructure (corresponding to the “X” or predictor variable) via EF (as indicated by the common EF score, corresponding to “M” or mediator variable). In this model, the overall relationship between white matter measures and motor performance is described by the “total effect” (c-path). The respective relationships between white matter measures and EF performance (a-path) and EF performance and motor performance (b-path) represent the “indirect effect” (ab-path), whereas the “direct effect” (c’-path) describes the remaining relationship between white matter measures and motor performance after accounting for EF performance. In other words, we hypothesized that EF performance significantly mediates the association between white matter measures and motor performance in older adults. This hypothesis entails that (a) white matter structure gives rise to brain function to support successful behavioral performance in the executive and in the motor domain, and (b) motor control relies on more generic, higher-order mental processes (i.e., executive functions) in addition to specific sensorimotor functions.

The mediation analysis was performed using the SPSS Process macro (v3.5.3) [82] in which 10,000 bootstrap samples were generated to determine the bias-corrected confidence interval (CI) for the indirect effect (ab-path) and thus mediation. Accordingly, an indirect effect with CI excluding zero was regarded as significantly mediating the relation between white matter micro- and macrostructure and motor performance via EF performance.

## RESULTS

### Associations between white matter measures and motor performance

To test the effect of white matter structural measures on motor performance, FDC, FD, and FC values for the eight transcallosal tracts were entered as predictors of BTT performance in a set of 24 regression analyses (Table 1). The results indicated that, after correcting for multiple comparisons, one standard deviation increase in M1-FDC and PF-FD significantly increased the BTT performance in older adults by 0.36 and 0.40 standard deviations, respectively. None of the FC-measures significantly predicted the BTT performance in older adults. Taken together, higher combined density and cross-section (FDC) of transcallosal fibers connecting bilateral motor cortices and higher fiber density (FD) of transcallosal tracts connecting bilateral prefrontal cortices were related to better performance of older adults in a complex motor task.

**Table 1:** Associations between white matter measures and motor performance.

**Transcallosal tract**	***β***	***t***	***p_Bonf. Corr._***
***Fiber density and cross-section (FDC)***			
orbitofrontal cortex (OF)	.19	1.69	> .999
prefrontal cortex (PF)	.37	3.15	.055
premotor and supplementary cortex (Pre/Supl)	.24	2.14	.084
**primary motor cortex (M1)**	**.36**	**3.52**	**.017**
primary sensory cortex (S1)	.26	2.48	.363
parietal cortex (Par)	.27	2.49	.356
temporal cortex (Temp)	.23	2.09	.945
occipital cortex (Occ)	.27	2.51	.338
***Fiber density (FD)***			
orbitofrontal cortex (OF)	.26	2.38	.469
**prefrontal cortex (PF)**	**.40**	**3.94**	**.004**
premotor and supplementary cortex (Pre/Supl)	.25	2.24	.665
primary motor cortex (M1)	.31	2.73	.186
primary sensory cortex (S1)	.32	2.77	.168
parietal cortex (Par)	.30	2.70	.202
temporal cortex (Temp)	.16	1.38	> .999
occipital cortex (Occ)	.29	2.56	.293
***Fiber cross-section (FC)***			
orbitofrontal cortex (OF)	-.00	-0.03	> .999
prefrontal cortex (PF)	.01	0.04	> .999
premotor and supplementary cortex (Pre/Supl)	.13	0.85	> .999
primary motor cortex (M1)	.42	2.91	.112
primary sensory cortex (S1)	.08	0.51	> .999
parietal cortex (Par)	.12	0.72	> .999
temporal cortex (Temp)	.27	1.94	> .999
occipital cortex (Occ)	.17	1.20	> .999

Note. *β* denotes the completely standardized regression coefficient. *t*, *t*-value. *p*-values are Bonferroni-corrected for multiple comparisons. Estimated total intracranial volume (TIV) was used as a nuisance variable of no interest in regressions.

### Associations between white matter measures and executive performance

To test the relationship between white matter structural measures and executive performance, FDC, FD, and FC values for the eight transcallosal tracts were entered as predictors of EF performance in a set of 24 regression analyses (Table 2). The results indicated that, after correcting for multiple comparisons, one standard deviation increase in Par-FDC, PF-FD, M1-FD and Par-FD significantly increased the EF performance in older adults by 0.35, 0.35, 0.38, and 0.43 standard deviations, respectively. None of the FC-measures significantly predicted the EF performance in older adults. Taken together, higher combined density and cross-section (FDC) of transcallosal fibers connecting bilateral parietal cortices was related to better executive abilities in older adults, as was higher density (FD) of transcallosal fibers connecting bilateral prefrontal, motor, and parietal cortices.

**Table 2:** Associations between white matter measures and executive functioning.

**Transcallosal tract**	***β***	***t***	***p_Bonf. Corr._***
***Fiber density and cross-section (FDC)***			
orbitofrontal cortex (OF)	.30	2.68	.216
prefrontal cortex (PF)	.25	2.06	> .999
premotor and supplementary cortex (Pre/Supl)	.27	2.47	.374
primary motor cortex (M1)	.29	2.68	.216
primary sensory cortex (S1)	.21	1.88	> .999
**parietal cortex (Par)**	**.35**	**3.23**	**.043**
temporal cortex (Temp)	.22	1.95	> .999
occipital cortex (Occ)	.31	2.87	.127
***Fiber density (FD)***			
orbitofrontal cortex (OF)	.33	3.05	.074
**prefrontal cortex (PF)**	**.35**	**3.29**	**.036**
premotor and supplementary cortex (Pre/Supl)	.34	3.08	.070
**primary motor cortex (M1)**	**.38**	**3.34**	**.031**
primary sensory cortex (S1)	.35	3.04	.077
**parietal cortex (Par)**	**.43**	**4.08**	**.002**
temporal cortex (Temp)	.26	2.28	.607
occipital cortex (Occ)	.25	2.12	.900
***Fiber cross-section (FC)***			
orbitofrontal cortex (OF)	.07	0.48	> .999
prefrontal cortex (PF)	-.25	-1.27	> .999
premotor and supplementary cortex (Pre/Supl)	.14	0.94	> .999
primary motor cortex (M1)	.12	0.82	> .999
primary sensory cortex (S1)	-.06	-0.33	> .999
parietal cortex (Par)	.18	1.10	> .999
temporal cortex (Temp)	.13	0.89	> .999
occipital cortex (Occ)	.34	2.45	.394

Note. *β* denotes the completely standardized regression coefficient. *t*, *t*-value. *p*-values are Bonferroni-corrected for multiple comparisons. Estimated total intracranial volume (TIV) was used as a nuisance variable of no interest in regressions.

### Associations between executive and motor performance

Simple regression analysis showed that common EF ability significantly predicted BTT performance in older adults (*β* = .46, *p* < .001). Thus, one standard deviation increase in executive functioning significantly increased motor performance in older adults by 0.46 standard deviations.

### Mediation analysis

**Figure 2:**
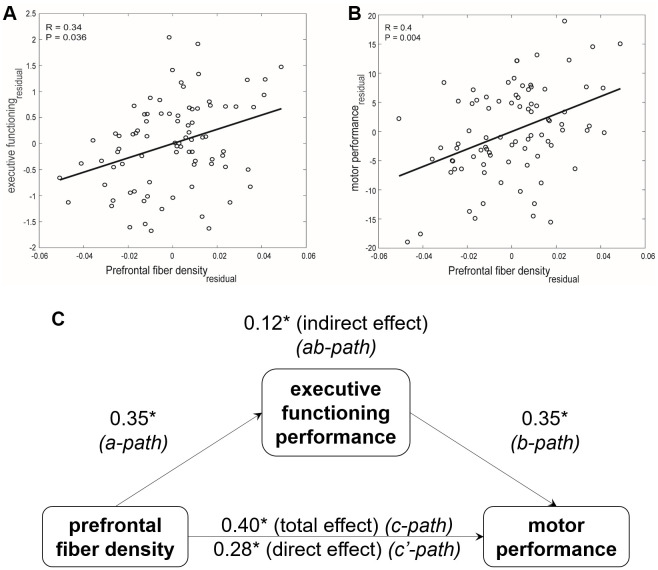
**Relationships between prefrontal fiber density, executive functioning performance, and motor performance in older adults.** Scatter plot and the best least square line for (**A**) executive functioning and (**B**) motor performance association with prefrontal fiber density, after accounting for the estimated total intracranial volume (TIV), are shown. Partial Pearson’s coefficients and the Bonferroni-corrected *p*-values are indicated. (**C**) Executive functioning performance mediates the relationship between prefrontal fiber density and complex motor performance in older adults. * *p* < 0.01.

The regression analyses for associations between white matter structural measures and motor performance (c-path) and associations between white matter structural measures and EF performance (a-path) revealed that only PF-FD showed significant associations with both EF performance (Figure 2A) and motor performance (Figure 2B) after correcting for multiple comparisons. To test whether EF performance significantly mediated the relationship between PF-FD and motor performance, a mediation model was run (Figure 2C). This analysis revealed a significant indirect effect of PF-FD on motor performance via EF performance (*β*_ab-path_ = .12, 95%-CI [.04, .22]). The direct effect of PF-FD on motor performance remained significant after accounting for EF performance (*β*_c’-path_ = .28, *p* = .007). Hence, EF performance partially mediated the effect of PF-FD on motor performance in older adults. In other words, EF partly accounts for the relationship between the density of transcallosal fibers connecting bilateral prefrontal cortices and complex motor control in older adults.

## DISCUSSION

We examined micro- and macrostructural properties of white matter tracts through the CC in older adults and related them to performance on tasks of EF and bimanual coordination. We found significant links between FD and FDC measures of transcallosal white matter structure in fronto-parietal and motor areas and executive as well as motor functioning. As hypothesized, EF significantly mediated the association between white matter connectivity and motor control. This effect was detectable for fiber density in CC tracts connecting bilateral prefrontal cortices.

### White matter measures and motor performance in older adults

Our analyses revealed that older adults who performed better on the complex bimanual task also had higher combined density and cross-section of CC fibers connecting the motor cortices. This finding is in line with the notion that successful bimanual control relies on efficient interhemispheric communication among primary motor areas. Specifically, transcallosal information exchange is thought to ensure a good balance between excitation and inhibition of contralateral brain activation during bimanual performance to suppress inadequate mirror movements [3, 12, 19]. Bilateral motor-cortical connectivity has also been found to be related to bimanual control tasks in earlier work [13, 16, 20]. Hence, this result adds to the existing literature by further corroborating the importance of white matter connectivity between primary motor areas in bimanual coordination in an older age group.

In addition, better bimanual task performance in older adults was related to higher fiber density of transcallosal tracts connecting left and right prefrontal cortices. This finding extends previous work from our group, where we found that age-associated alterations in bimanual performance were mediated by micro- and macrostructural properties of the genu of the CC, i.e., fibers connecting left and right prefrontal cortices [14]. Together with our current data, those findings point toward a potential link between white matter connectivity of prefrontal cortices, bimanual coordination, and EF, because executive abilities are typically attributed to prefrontal portions of the brain. In support of this perspective, involvement of prefrontal brain regions in the performance of complex motor coordination tasks has also been demonstrated by functional neuroimaging work [29, 30, 83], potentially reflecting increased recruitment of cognitive resources in older adults as compensation for age-associated functional decline [2, 84, 85]. Prefrontal brain areas have also been linked to learning in bimanual coordination. Specifically, microstructural properties of transcallosal fibers connecting the prefrontal cortices have been found to predict more successful bimanual motor learning in young adults [18]. In older adults, increased recruitment of prefrontal brain regions during early learning of a complex bimanual coordination task was predictive of performance improvement on that task [86]. Hence, findings from functional and diffusion MRI studies converge in that they consistently suggest the involvement of prefrontal brain areas in bimanual motor tasks, both for task performance and for learning. Such prefrontal involvement might be particularly important to preserve motor functions in older adults.

### White matter measures and EF in older adults

We also found relationships between microstructural properties of transcallosal tracts and EF in older adults. Specifically, better executive performance was associated with higher fiber density of tracts connecting bilateral prefrontal, motor, and parietal cortices. For white matter tracts between left and right parietal areas, the combined fiber density and cross-section also significantly predicted EF performance. Hence, EF was associated with white matter structure in fronto-parietal areas. This is in line with earlier findings of links between EF and transcallosal fronto-parietal white matter connections [42, 44]. In addition, interhemispheric frontal connections as well as a fronto-parietal subnetwork have been suggested to form a substantial portion of a structural executive network [31]. This notion is also supported by functional neuroimaging work, suggesting fronto-parietal brain areas to constitute functional networks that are crucial for intact EF [45]. Our findings of significant associations between EF and fronto-parietal white matter micro- and macrostructure in older adults are thus consistent with earlier work.

We also found an association between EF and CC-tracts connecting primary motor cortices. This finding might point toward a general involvement of higher cognitive functions in interhemispheric motor coordination [3, 19]. While it is possible that such an interrelation is limited to older adults, who may rely on cognitive resources to a larger extent than young adults during bimanual control, general aging effects of associations between EF and motor areas remain to be investigated in lifespan samples. One might argue that the relationship between EF performance and M1-M1 connectivity reflects motor demands of the EF tasks used in the present study. However, we can rule out this possibility because we assessed EF using a latent variable that is reflective of the commonalities among all EF tasks under investigation. As the response modes for the different EF tasks were rather diverse, our EF metric is unlikely to contain a large portion of variability related to motor demands.

### Motor and executive performance in older adults

As in our previous work, we found a significant relationship between EF and motor performance in older adults [28]. While this result is based on a subsample reported in our earlier paper and therefore cannot be interpreted as independent evidence for a link between EF and motor performance, it is well in line with earlier findings [26, 27] as well as the proposed theoretical link between EF and bimanual coordination [3, 19].

### EF performance mediates the association between prefrontal fiber density and motor performance in older adults

In line with our hypothesis, we found the relationship between transcallosal white matter structure and complex motor control to be mediated by EF in older adults. In other words, EF performance partly accounted for the relationship between interhemispheric white matter connectivity of bilateral prefrontal cortices and motor performance. One potential interpretation of this pattern is that a set of white matter connections forms the structural basis for both EF and motor control. At the same time, executive functions (i.e., higher-level mental processes) guide and govern bimanual coordination (i.e., lower-level mental processes). Decreased white matter structure in prefrontal interhemispheric connections might hamper motor performance—both directly, by a degree of “disconnection” of the structural basis for motor control, but also indirectly via decreased EF, by a degree of “disconnection” of the structural basis for executive processes. Consequently, reliance on EF is hampered during the performance of a motor task, which might exacerbate motor functioning decline (that, in turn, is partly attributable to decreased white matter connectivity). Hence, the role of EF should be taken into account when assessing the relationship between white matter structure and complex motor performance. This may be particularly relevant for older populations, who appear to rely increasingly on executive functions when performing challenging motor tasks [29, 30].

### Considerations on the white matter measures

Our results generally showed relationships between performance measures and FD- and FDC-metrics, but we did not observe significant brain-behavior associations for FC-metrics. Unlike FD, which is reflective of microstructural properties within a given voxel, FC is sensitive to macrostructural characteristics of a fiber bundle. Significant relationships between performance and FDC-metrics indicate that FD and FC combined show associations with behavior. For the majority of the tracts, the relationship between performance indicators and FC-metrics were still numerically positive, albeit not statistically significant.

### Limitations and outlook

The following limitations should be taken into account when interpreting the present results. First, we focused exclusively on a cohort of older adults in the present study in order to investigate interrelations between white matter micro- and macrostructure, EF, and bimanual control within this group. Therefore, we cannot derive conclusions regarding the aging process per se, which would require a lifespan sample and preferably a longitudinal design [87, 88]. However, the present data broaden our understanding of the importance of EF for complex motor control in older adults in that they support the notion that older adults might increasingly rely on EF when performing motor tasks [29, 30]. In addition, they corroborate the hypothesized EF involvement in the link between white matter structure and bimanual control in older adults—an idea that was raised by findings of age-related variations in anterior CC-metrics as contributors to decreased bimanual performance [14].

Second, the correlational nature of this dataset warrants careful interpretation. Specifically, these results do not provide insights into the process of executive involvement in bimanual control in a causal or in a mechanistic sense [89]. Such relationships could be targeted with interventional designs, e.g., by experimentally improving EF through training interventions and assessing training-induced changes in motor outcomes.

Third, we solely focused on tracts of the CC in this study. This choice was motivated by the fact that the CC is the brain’s largest white matter structure with a crucial role in bimanual coordination [12]. Additionally, we aimed to follow up on earlier findings of anterior CC-involvement in age-related bimanual coordination changes by assessing these brain-motor associations in conjunction with individual differences in EF performance [14]. However, it should be emphasized that successful bimanual coordination is a multifactorial process that involves a variety of brain areas depending on task complexity and the context in which tasks are performed, including the available sensory information [19]. Future work might therefore expand our analyses by examining executive-motor associations with white matter tracts other than the CC. For instance, given the role of the basal ganglia and the cerebellum in both executive and motor performance, fronto-striatal and fronto-cerebellar tracts represent potential target structures for further investigation.

## CONCLUSIONS

The present results contribute to our understanding of age-associated motor functioning decline. Specifically, they shed light on how the control of complex movements is associated with both white matter connectivity and executive abilities in older adults. They support the notion that age-associated changes in white matter connectivity could negatively affect motor functioning both directly, but also indirectly via executive functioning decline. Hence, executive abilities should be considered when investigating brain-motor associations in older adults and in the design of interventions to preserve motor control. Future work should aim to further investigate the role of EF in motor control by the implementation of training protocols in longitudinal intervention studies.

## Supplementary Material

Supplementary Figures

Supplementary Tables
