# Supplementary material for: Bridging cognition and action: executive functioning mediates the relationship between white matter fiber density and complex motor abilities in older adults
Source: Aging (Albany NY). 2022 Aug 22;14(18):7263–81. doi: 10.18632/aging.204237 (PMC9550248; doi:10.18632/aging.204237)
Supplement: Supplementary Figures [file aging-14-204237-s001.pdf]

SUPPLEMENTARY FIGURES

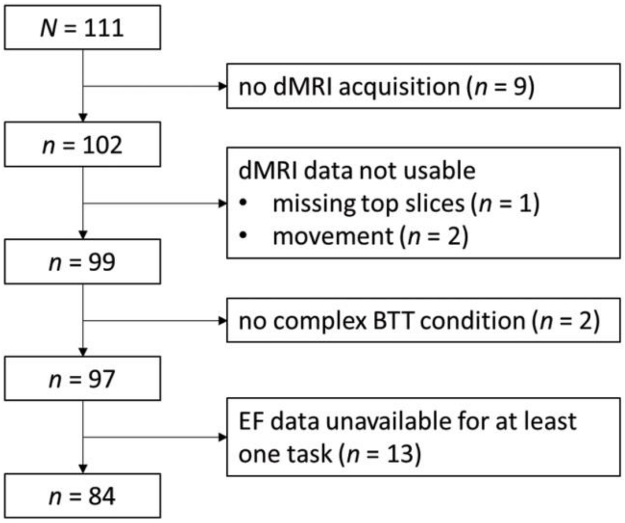

Supplementary Figure 1. Flow chart for selection of observations. *Note.* Only complete datasets were retained for the current analyses. Please see Seer et al. (2021) for details regarding the availability of EF data. BTT = bimanual tracking task, dMRI = diffusion magnetic resonance imaging, EF = executive functioning.

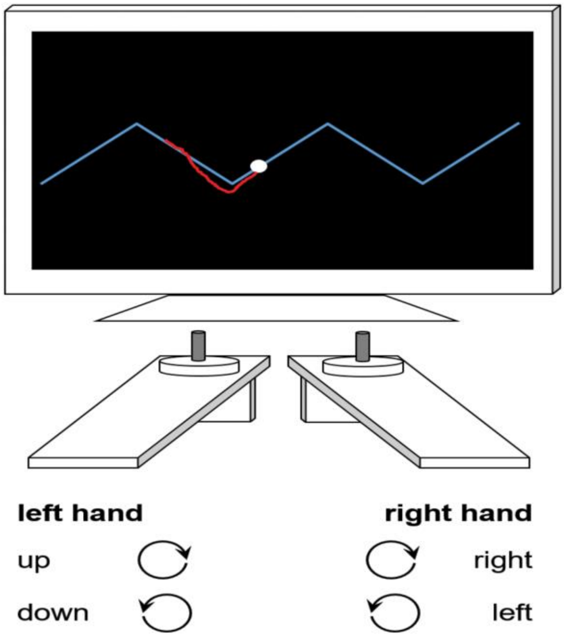

Supplementary Figure 2. Setup of the bimanual tracking task (BTT).
