# Supplementary material for: Bridging cognition and action: executive functioning mediates the relationship between white matter fiber density and complex motor abilities in older adults
Source: Aging (Albany NY). 2022 Aug 22;14(18):7263–81. doi: 10.18632/aging.204237 (PMC9550248; doi:10.18632/aging.204237)
Supplement: Supplementary Tables [file aging-14-204237-s002.pdf]

SUPPLEMENTARY TABLES

Supplementary Table 1. Outcome measures for the executive functioning tasks.

Task	Outcome
Inhibition	
Antisaccade	proportion correct responses in antisaccade blocks
Number Stroop	median RT for correct responses for incongruent minus congruent trials
Stop Signal	difference between the median RT on go-trials in mixed blocks and mean stop-signal delay (averaged across stop trials) (= stop-signal RT)
Shifting	
Category Switch	difference of median RT between switch and repeat trials
Color-Shape	difference of median RT between switch and repeat trials
Number-Letter	difference of median RT between switch and repeat trials
Updating	
Digit Span	total number of correct trials
Keep Track	proportion of correctly recalled items
Spatial 2-Back	proportion of correct responses

Note. RT, reaction time.

Supplementary Table 2. Descriptive statistics for the white matter measures.

	M	*SD*	IQ25	IQ50	IQ75	min	max
FDC							
OF	0.39	0.05	0.36	0.39	0.42	0.30	0.55
PF	0.30	0.03	0.28	0.29	0.32	0.25	0.39
Pre/Supl	0.43	0.05	0.39	0.42	0.45	0.32	0.60
M1	0.42	0.05	0.39	0.42	0.45	0.33	0.57
S1	0.37	0.04	0.34	0.37	0.40	0.28	0.46
Par	0.36	0.05	0.33	0.36	0.39	0.24	0.46
Temp	0.50	0.05	0.46	0.49	0.54	0.38	0.61
Occ	0.44	0.05	0.41	0.44	0.47	0.32	0.62
FD							
OF	0.38	0.03	0.37	0.39	0.40	0.29	0.44
PF	0.30	0.02	0.29	0.30	0.32	0.24	0.35
Pre/Supl	0.41	0.03	0.39	0.41	0.43	0.33	0.50
M1	0.40	0.03	0.39	0.41	0.42	0.31	0.46
S1	0.37	0.03	0.36	0.38	0.39	0.27	0.42
Par	0.36	0.03	0.34	0.36	0.38	0.27	0.41
Temp	0.48	0.03	0.47	0.49	0.50	0.39	0.53
Occ	0.41	0.03	0.40	0.42	0.43	0.30	0.49
FC							
OF	0.02	0.09	-0.05	0.02	0.07	-0.19	0.29
PF	-0.01	0.09	-0.08	-0.02	0.05	-0.21	0.21
Pre/Supl	0.02	0.09	-0.03	0.02	0.06	-0.19	0.25
M1	0.04	0.08	-0.01	0.04	0.08	-0.14	0.21
S1	-0.01	0.07	-0.05	-0.01	0.03	-0.18	0.18
Par	-0.01	0.10	-0.08	-0.01	0.04	-0.25	0.31
Temp	0.02	0.08	-0.03	0.03	0.09	-0.18	0.23
Occ	0.07	0.09	-0.01	0.07	0.13	-0.10	0.29

Note. Values for FC were log transformed. FDC, combined fiber density and cross-section; FD, fiber density; FC, fiber cross-section; OF, orbitofrontal cortex; PF, prefrontal cortex; Pre/Supl, premotor and supplementary cortex; M1, primary motor cortex; S1, primary sensory cortex; Par, parietal cortex; Temp, temporal cortex; Occ, occipital cortex.
